# The influence of maternal mental illness on vaccination uptake in children: a UK population-based cohort study

**DOI:** 10.1007/s10654-020-00632-5

**Published:** 2020-04-24

**Authors:** Cemre Su Osam, Matthias Pierce, Holly Hope, Darren M. Ashcroft, Kathryn M. Abel

**Affiliations:** 1 Centre for Women’s Mental Health, School of Health Sciences, Faculty of Biology, Medicine and Health, University of Manchester, Jean McFarlane Building, Oxford Road, Manchester, M13 9PL UK; 2 Centre for Pharmacoepidemiology and Drug Safety, School of Health Sciences, Faculty of Biology, Medicine and Health, University of Manchester, Stopford Building, Oxford Road, Manchester, M13 9PT UK; 3 NIHR Greater Manchester Patient Safety Translational Research Centre (PSTRC), University of Manchester, Manchester, M13 9PL UK; 4 Greater Manchester Mental Health NHS Foundation Trust, Bury New Road, Prestwich, Manchester, Greater Manchester M25 3BL UK

**Keywords:** Maternal mental illness, Child vaccination, Primary care, CPRD

## Abstract

Reduced vaccination uptake is a growing and global public health concern. There is limited knowledge about the effect of maternal mental illness (MMI) on rates of childhood vaccination. This retrospective cohort study examined 479,949 mother-baby pairs born between 1993 and 2015 in the Clinical Practice Research Datalink (CPRD GOLD), a UK-based, primary health-care database. The influence of MMI on children’s vaccination status at two and five years of age was investigated using logistic regression adjusting for sex of the child, child ethnicity, delivery year, maternal age, practice level deprivation quintile and region. The vaccinations were: 5-in-1 (DTaP/IPV/Hib) and first dose MMR by the age of two; and all three doses of 5-in-1, first and second dose of MMR vaccines by the age of five. Exposure to MMI was defined using recorded clinical events for: depression, anxiety, psychosis, eating disorder, personality disorder and alcohol and substance misuse disorders. The likelihood that a child completed their recommended vaccinations by the age of two and five was significantly lower among children with MMI compared to children with mothers without mental illness [adjusted odds ratio (aOR) 0.86, 95% CI 0.84–0.88, *p* < 0.001]. The strongest effect was observed for children exposed to maternal alcohol or substance misuse (at two years aOR 0.50, 95% CI 0.44–0.58, *p* < 0.001). In the UK, an estimated five thousand more children per year would be vaccinated if children with MMI had the same vaccination rates as children with well mothers. Maternal mental illness is a hitherto largely unrecognised reason that children may be missing vital vaccinations at two and five years of age. This risk is highest for those children living with maternal alcohol or substance misuse.

**Electronic supplementary material:**

The online version of this article (10.1007/s10654-020-00632-5) contains supplementary material, which is available to authorized users.

## Introduction

Despite the fact that in most countries vaccines are provided free at the point of access [1], 15% of children remain unvaccinated globally [2]. Recent outbreaks of measles in Europe [3, 4] and the US [5] indicate a significant decline in herd immunity as a result of reduced vaccination uptake in infants. Such trends of reducing childhood vaccination have been attributed to scepticism about the safety of vaccination, particularly following the falsified report by Wakefield [6] linking the Mumps, Measles and Rubella (MMR) vaccine with childhood autism.

People with mental illness are less likely to benefit from public health information campaigns [7, 8] but it is unclear whether mothers with mental illness are more or less influenced by messages about vaccination safety. This is important because mothers are usually the primary caregivers and take a central role in their children’s health [9] so the extent to which their children access preventative healthcare is of public health concern. Recently, using presence or absence of maternal mental illness (MMI) from primary care records, we estimated that almost one in four children in the UK has a mother with a mental illness [10] and this number may be growing. These estimates indicate the prevalence of children exposed to different types of MMI in the UK as: non-affective psychosis 0.2%; affective psychosis 0.3%, depression as 18.4%, anxiety as 7.9%; eating disorders 0.1%; personality disorder 0.1% and alcohol and substance misuse as 0.3% [10].

There is limited evidence about the extent to which MMI influences rates of vaccination uptake. Existing studies report associations between decreased vaccination uptake, maternal depression [11, 12] and maternal psychotic disorder [13]. However, these rely on small samples (< 5000), mostly use maternal self-report of mental illness and were ascertained more than a decade ago. If children with MMI remain at risk of significantly lower vaccination uptake, this represents an important public health concern in a group already known to be multiply deprived [14] and vulnerable to premature mortality of treatable cause [15, 16]. We addressed this outstanding knowledge gap in a large, contemporary and high-quality population-based primary care cohort.

We hypothesised first that children with MMI would receive fewer vaccinations overall than children with healthy mothers. Second, we hypothesised that offspring of mothers with psychotic disorders would be significantly less likely to receive vaccination compared to mothers with common mental illnesses (i.e. depression and anxiety). Finally, we explore whether any effect that MMI has on vaccination rate has changed over time, and in the period including the MMR scandal.

## Methods

### Data source

Data for this retrospective cohort study were delineated from the Clinical Practice Research Datalink (CPRD GOLD) which contains anonymised electronic health records from 10% of UK general practices and is considered demographically representative of the UK population [17]. CPRD holds data on clinical consultations, immunisations, prescriptions and external healthcare referrals. Data entries are made by general practitioners (GPs) using the Read Code [18] framework.

The cohort was identified from the CPRD mother-baby link; a previously developed algorithm [19] which identifies birth records for women and pairs those to babies if: the baby is registered at the same general practice as the mother; the baby’s birthday is within 60 days of delivery date and the baby shares a specific family identifier based on residential address [20]. We also linked our cohort with Hospital Episode Statistics (HES) dataset to obtain additional information on the child’s ethnicity.

### Cohort selection

**Fig. 1:**
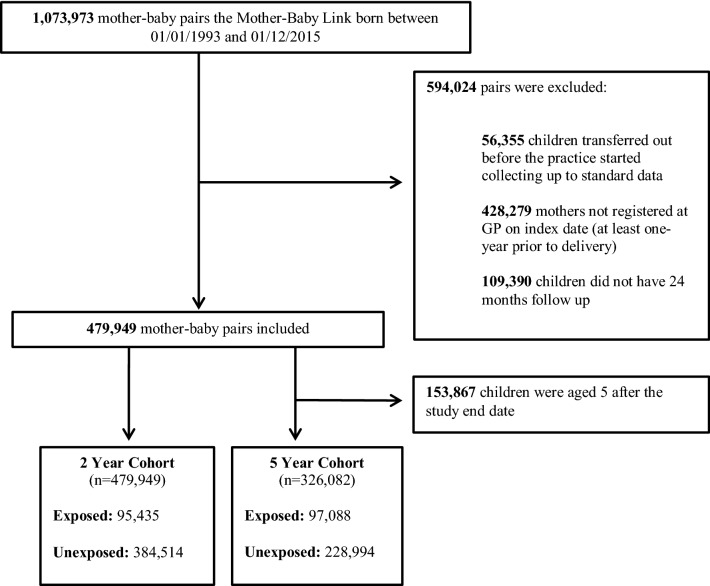
CONSORT diagram of the mother-baby cohort selection process

The cohort included a sample of children identified from the CPRD’s Mother-Baby Link born between 1st January 1993 and 31st December 2015. Follow-up was defined from the date of birth until the earliest date that the child transferred out of the practice, died or the practice stopped contributing data to CPRD. Children were excluded from the cohort if: they were born before the participating general practice fulfilled the minimum data quality standards set by the CPRD; their mother had not been registered at a participating general practice for at least one year prior to birth; or if they did not have a minimum of two years follow-up (Fig. 1).

Two separate cohorts were constructed for children with complete follow-up data until age two and until age five years to reflect when children are vaccinated. The scheduled times for vaccination in this study are four, 13 and 40 months; therefore, the two and five years time points allow for delays in vaccination for legitimate reasons not associated with MMI (e.g. child infections). Moreover, these time points allow this study to be comparable with childhood vaccination statistics in different regions of the UK [21–24].

### Maternal mental illness

Children were defined as exposed to MMI if their mother had a clinical event indicating mental illness between one year prior to birth up to the age of two (for the two years cohort) and five (for the five years cohort) as previously described [10]. Since children can exist in both cohorts, some of the following exposure categories may overlap. Mental illness was defined using the following categories (mapped to ICD-10 codes): (1) *psychotic disorders* (ICD-10: F20–4, F25, F28–9, F30–31), (2) *depressive disorders* (F32–9), (3) *anxiety and stress*-*related disorders* (F40–48), (4) *eating disorders* (F50), (5) *personality disorders* (F60–3, F67–9) and (6) *alcohol/drug dependence* (F10–16, F18–19). Final code lists used are available at www.clinicalcodes.org.

A recorded mental illness diagnosis by the GP was considered sufficient to identify mothers with mental illness. However, in the UK, there is an increasing preference among GPs to record mental illness symptoms instead of diagnostic labels [25]. Therefore, we adapted the algorithm created by Abel et al. [10] which identifies mental illness if a mother has a symptom within three months of a prescription for medication such as antidepressants, antipsychotics and anxiolytics that has a recognised indication for the treatment of the same mental illness. The medications used and the relevant symptom-categories were antidepressants (depressive disorder); antipsychotics (non-affective psychosis), anxiolytics/hypnotics (anxiety disorders), mood stabilisers (affective psychosis); and drugs used to treat substance dependence.

### Childhood vaccinations

We only selected vaccinations which have not changed during the study period, in the number of doses or child age at administration. These vaccinations were: three doses of 5-in-1; two doses of MMR; and one dose of 4-in-1 preschool booster (Table 1).

**Table 1:** Childhood vaccinations examined in the study

Vaccine (active ingredients)	Given at age (in months)	Investigated for age-group (in years) for this study
5-in-1 (DTaP/IPV/Hib) _diphtheria, tetanus, acellular pertussis, inactivated polio vaccine and hib (haemophilus influenzae type b)_	2, 3, 4	2
MMR _measles, mumps and rubella_	13 ^(1st dose)^, 40–60 ^(2nd dose)^	2, 5
4-in-1 (DTaP/IPV) _diphtheria, tetanus, acellular pertussis, inactivated polio vaccine_	40	5

Dichotomous variables were created for each child indicating whether they had a recorded dose of all the necessary vaccinations or not (yes/no) by the specified age by using the date of the vaccination recorded in the child’s immunisation files. Children were recorded as having received all vaccinations if they had three doses of 5-in-1 and one dose of MMR by age two. Therefore, children aged five, were counted as “received all vaccinations” only if they had received all necessary vaccinations by age two as well as second dose of MMR and one dose of 4-in-1.

The proportion of those with recorded 4-in-1 was only around 10% which was considered unfeasibly low. This has been reported previously by Public Health England and the National Health Service [26, 27] and it has been suggested that this relates to many children receiving 4-in-1 outside the recommended timeframe, or because some children only receive 3-in-1. Therefore, we assumed that data were not well recorded for this vaccine and excluded it from the analysis.

### Covariates

Demographic data were extracted on maternal age at birth, child sex and geographical UK region of the general practice (categorised as: North East, North West, Yorkshire & The Humber, East Midlands, West Midlands, East of England, South West, South Central, London, South East Coast, Northern Ireland, Scotland and Wales). Child ethnicity (categorised as: Asian, Black, Mixed and White) was extracted from CPRD and HES. We prioritised HES data on ethnicity when data were available from both sources because HES data has been previously validated using other linked data sources [28]. The quintile of the Index of Multiple Deprivations (IMD) illustrates the deprivation at the local area level, based on employment, income, education, health and disability and crime, housing and the lived environment. These areas are rated and divided into quintiles from least to most deprived areas. The IMD of the registered practices was extracted based on their postcodes. The number of face-to-face GP appointments of the mothers nine months prior to birth was calculated with an aim to create a proxy for maternal engagement in primary healthcare services.

### Analysis

The association between MMI and vaccination uptake was estimated using odds ratios calculated using logistic regression models. The first model adjusted for variables thought potentially to confound the relationship between MMI and childhood vaccination status: sex of the child, child ethnicity, delivery year, maternal age, practice level deprivation quintile and geographical region. In the second model we included, as an additional variable, the count of GP appointments nine months prior to birth to investigate whether part of the associations could be explained by maternal engagement in primary care services. We estimated how many extra children would be vaccinated over the study period (19 years) if children with MMI had the same vaccination rates as children with healthy mothers using the formula:

$$\mathop \sum \limits_{y = 1998}^{2017} LB_{y - 5} *\pi_{y} *RD_{y}$$

where $\pi_{y}$ is the prevalence of MMI estimated in children aged five years in year *y*; LB_y−5_ is the number of live births in the five years prior, provided from national statistics [29–31]; and RD is the estimated difference in vaccination rate between those with MMI and those without, in year y. Confidence intervals for this estimate were calculated using the bootstrap procedure [32], using the normal approximation over 10,000 bootstrap samples.

In order to investigate whether the effect of MMI on vaccination has changed over the period covering the MMR scandal, adjusted odds ratio were calculated for each analysis year (1993–2015) using an interaction term between year and MMI.

In all regression analyses, continuous variables were centred and a squared term was included; Wald-test statistics were used to determine 95% confidence intervals and *p* values and Chi square test was used to test the interaction. Clustering by maternal siblings was dealt with by calculating the standard errors using the Hubber/White sandwich estimator [33]. Data were analysed using Stata SE 15.0.

### Sensitivity analyses

Data recording in the CPRD has significantly improved from 2005 as a result of the introduction of the Quality and Outcomes Framework (QOF) in 2004 [34]. Therefore, a sensitivity analysis was conducted to investigate whether or not improvements in data recording affected results by repeating the analysis excluding those born before 2005. We also anticipated that some children may have registered to their participating practices later than age two even if their mothers were fully registered at the same practice. Vaccination records are back-dated when a child is registered at a general practice; however, we were concerned about the quality of vaccination history records for these late registered children. Therefore, we conducted a second sensitivity analysis to investigate the potential effects of late registered children by re-running the analyses excluding this group.

## Results

The study cohort consisted of 479,949 mother-baby pairs in the cohort followed-up to two years old (the two-year cohort) and 326,082 pairs followed-up to five years old (the five-year cohort, Fig. 1). The mean maternal age at delivery was 30.3 years (standard deviation = 5.8) and 48.8% of children were female (Table 2). Children exposed to MMI were more likely than those without to be registered at a general practice in the lowest deprivation quintile (30.7% vs 25.5%).

**Table 2:** Cohort demographics for the two and five years cohorts, by maternal mental illness status

	2 Year cohort (n = 479,949)				5 Year cohort (n = 326,082)			
	Children with maternal mental illness (n = 95,435)	%	Children without maternal mental illness (n = 384,514)	%	Children with maternal mental illness (n = 97,088)	%	Children without maternal mental illness (n = 228,994)	%
**Child gender**								
Female	46,299	48.5	188,058	48.9	47,229	48.7	111,951	48.9
Male	49,136	51.5	196,456	51.1	49,859	51.4	117,043	51.1
**Child ethnicity**								
Asian/British Asian	1279	1.3	13,679	3.6	1339	1.4	7515	3.3
Black/black British	604	0.6	5991	1.6	549	0.6	3026	1.3
Mixed	1591	1.7	7021	1.8	1336	1.4	3386	1.5
Other	511	0.5	3565	0.9	474	0.5	1876	0.8
White	62,479	65.5	236,265	61.5	62,740	64.6	139,957	61.1
Unknown	28,971	30.4	117,993	30.7	30,650	31.6	73,234	32.0
**Maternal age at delivery**								
< 19	4959	5.2	12,370	3.2	5140	5.3	6761	3.0
20–24	17,996	18.9	47,759	12.4	17,236	17.8	26,457	11.6
25–29	26,098	27.4	95,669	24.9	26,476	27.3	55,581	24.3
30–34	27,081	28.4	129,771	33.8	28,247	29.1	79,267	34.6
35–39	15,473	16.2	79,789	20.8	16,207	16.7	49,471	21.6
40 >	3828	4.0	19,156	5.0	3782	3.9	11,457	5.0
**Maternal mental illness**								
Any mental illness^a^	95,435	19.9	–	–	97,088	29.8	–	–
Psychotic disorder	1120	0.3	–	–	1319	0.6	–	–
Depressive disorder	78,062	16.9	–	–	79,355	25.7	–	–
Anxiety disorder	28,932	7.0	–	–	36,744	13.8	–	–
Eating disorder	854	0.2	–	–	1124	0.5	–	–
Personality disorder	500	0.1	–	–	594	0.3	–	–
Substance and alcohol abuse	1549	0.4	–	–	1943	0.8	–	–
**UK IMD quintile**_based on GP location_								
1 least deprived	14,085	14.8	72,719	18.9	15,347	15.8	46,276	20.2
2	14,753	15.5	62,129	16.2	14,847	15.3	36,738	16.0
3	17,232	18.1	72,012	18.7	17,391	17.9	42,489	18.6
4	20,045	21.0	79,491	20.7	20,240	20.9	46,708	20.4
5 most deprived	29,320	30.7	98,163	25.5	29,263	30.1	56,783	24.8
**Region**								
North East	1992	2.1	7280	1.9	2029	2.1	4388	1.9
North West	13,323	14.0	46,544	12.1	13,811	14.2	28,979	12.7
Yorkshire & The Humber	3137	3.3	13,693	3.6	3148	3.2	8144	3.6
East Midlands	4222	4.4	14,688	3.8	4074	4.2	8433	3.7
West Midlands	8886	9.3	36,298	9.4	8987	9.3	21,911	9.6
East of England	7536	7.9	35,376	9.2	7573	7.8	20,536	9.0
South West	8747	9.2	33,305	8.7	8829	9.1	19,479	8.5
South Central	10,755	11.3	44,153	11.5	10,657	11.0	26,391	11.5
London	5641	5.9	36,529	9.5	5386	5.6	19,093	8.3
South East Coast	8021	8.4	37,712	9.8	8037	8.3	22,506	9.8
Northern Ireland	4813	5.0	13,370	3.5	5193	5.4	8328	3.6
Scotland	9222	9.7	31,700	8.2	9401	9.7	18,906	8.3
Wales	9140	9.6	33,866	8.8	9963	10.3	21,900	9.6

^a^Note that maternal illness categories are not mutually exclusive

Twenty percent of children (95,435/479,949) in the two years cohort and 29.8% of children (97,088/326,082) in the five years cohort had a mother with a mental illness recorded between one year prior to their birth up to their second and fifth birthday, respectively. The most common disorder was maternal depression (25.7% for the five years cohort); less than 1% had a diagnosis of psychotic disorder.

### Maternal mental illness and vaccination uptake

#### At two years

Across the study period between 1993 and 2015, the proportion of children with healthy mothers who had all their necessary vaccinations at two years was 88.0%; for children with MMI, it was 86.3% (Table 3).

**Table 3:** Association between receiving vaccinations at age two and five years and maternal mental illness

Up to date vaccinations at	Number vaccinated (%)	Number not vaccinated (%)	Unadjusted model		Adjusted model-1^a^		Adjusted model-2^b^	
			OR (95% CI)	*p* value	OR (95% CI)^a^	*p* value	OR (95% CI)^b^	*p* value
**2 Year**								
Unexposed to maternal mental illness	338,398 (88.0)	46,116 (12.0)	REF		REF		REF	
Exposed to any maternal mental illness	82,361 (86.3)	13,074 (13.7)	0.86 (0.84–0.88)	< 0.001	0.86 (0.84–0.88)	< 0.001	0.86 (0.84–0.88)	< 0.001
Psychotic disorder	973 (86.9)	147 (13.1)	0.90 (0.75–1.08)	0.267	0.86 (0.71–1.03)	0.103	0.85 (0.70–1.02)	0.088
Depressive disorder	67,241 (86.1)	10,821 (13.9)	0.85 (0.83–0.87)	< 0.001	0.86 (0.84–0.88)	< 0.001	0.86 (0.84–0.88)	< 0.001
Anxiety disorder	25,052 (86.6)	3880 (13.4)	0.88 (0.85–0.91)	< 0.001	0.86 (0.83–0.89)	< 0.001	0.86 (0.82–0.89)	< 0.001
Eating disorder	738 (86.4)	116 (13.6)	0.87 (0.71–1.06)	0.174	0.94 (0.77–1.16)	0.579	0.94 (0.76–1.15)	0.533
Personality disorder	430 (86.0)	70 (14.0)	0.84 (0.65–1.09)	0.181	0.75 (0.58–0.98)	0.037	0.74 (0.57–0.97)	0.029
Substance and alcohol abuse	1218 (78.6)	331 (21.4)	0.50 (0.44–0.57)	< 0.001	0.50 (0.44–0.58)	< 0.001	0.50 (0.44–0.57)	< 0.001
**5 Year**								
Unexposed to maternal mental illness	188,399 (82.3)	40,595 (17.7)	REF		REF		REF	
Exposed to any maternal mental illness	77,569 (79.9)	19,519 (20.1)	0.86 (0.84–0.87)	< 0.001	0.86 (0.84–0.88)	< 0.001	0.85 (0.84–0.87)	< 0.001
Psychotic disorder	1024 (77.6)	295 (22.4)	0.75 (0.65–0.86)	< 0.001	0.71 (0.62–0.82)	< 0.001	0.71 (0.61–0.81)	< 0.001
Depressive disorder	63,302 (79.8)	16,053 (20.2)	0.85 (0.83–0.87)	< 0.001	0.86 (0.84–0.88)	< 0.001	0.85 (0.83–0.87)	< 0.001
Anxiety disorder	29,291 (79.7)	7453 (20.3)	0.85 (0.82–0.87)	< 0.001	0.84 (0.82–0.87)	< 0.001	0.84 (0.81–0.86)	< 0.001
Eating disorder	884 (78.7)	240 (21.4)	0.79 (0.68–0.93)	0.004	0.83 (0.71–0.98)	0.024	0.83 (0.71–0.99)	0.018
Personality disorder	454 (76.4)	140 (23.6)	0.70 (0.57–0.85)	< 0.001	0.64 (0.52–0.78)	< 0.001	0.63 (0.51–0.77)	< 0.001
Substance and alcohol abuse	1354 (70.0)	580 (30.0)	0.50 (0.45–0.56)	< 0.001	0.50 (0.45–0.56)	< 0.001	0.50 (0.45–0.55)	< 0.001

^a^Adjusted for sex of the child, child ethnicity delivery year, maternal age, practice level deprivation quintile and region

^b^Adjusted for all variables in model one, plus number of GP visits nine months prior to the birth

See supplementary Tables 4 and 5 for estimates relating to adjusted variables

After adjusting for sex of the child, child ethnicity, delivery year, maternal age, practice level deprivation quintile and region, the likelihood that children received both their 5-in-1 and MMR 1st dose by age of two years was estimated to be 14% lower in children with MMI, compared to children with healthy mothers (aOR 0.86, 95% CI 0.84–0.88). Children with maternal substance or alcohol misuse disorders were half as likely to receive these compared to unexposed children (aOR 0.50, 95% CI 0.44–0.58).

#### At five years

At age of five years, the proportion of children with healthy mothers who received all necessary vaccinations was 82.3% and for children with MMI, it was 79.9% (Table 3).

After adjusting for sex of the child, child ethnicity, delivery year, maternal age, practice level deprivation quintile and region, children exposed to any MMI had significantly reduced likelihood of receiving all three vaccinations of 5-in-1, MMR 1st and MMR 2nd dose at five years (aOR 0.86, 95% CI 0.84–0.88). Mothers with a psychotic disorder were 29% less likely to vaccinate their children across the complete vaccination programme by age of five (aOR 0.71, 95% CI 0.62–0.82). Including the count of mother’s GP appointments nine months prior to delivery in the models, for both the two and five years cohorts, did not substantively alter results (Table 3).

If children with MMI had the same vaccination rate as children with healthy mothers then, on average 5010 additional children per year would have been vaccinated by age five (Table 4).

**Table 4:** Number of children who would have been vaccinated if children with any maternal mental illness had the same vaccine uptake as children without

Year	Live birth rates in the UK^a^	MMI prevalence rate in 5 years cohort (%)	Vaccination rate in children with MMI (%)	Vaccination rate in children with well mothers (%)	Number of extra children would have been vaccinated in 5 years cohort
1998	761,526	27	76	72	8888
1999	750,480	28	77	73	8225
2000	731,882	28	76	74	5923
2001	733,163	30	75	72	4807
2002	726,622	30	76	74	4554
2003	716,888	31	78	75	4952
2004	699,976	31	76	72	8430
2005	679,029	32	75	73	5446
2006	669,123	31	75	73	3360
2007	668,777	32	76	74	5725
2008	695,549	31	79	76	5451
2009	715,996	31	81	81	1348
2010	722,549	30	85	83	3798
2011	748,563	29	85	81	7841
2012	772,245	29	87	85	5148
2013	794,383	28	89	86	4816
2014	790,204	29	89	88	4003
2015	807,271	29	89	89	2114
2016	807,776	29	90	89	4239
2017	812,970	28	90	90	1125
Total (95% CI)					100,193 (85,903–114,482)

^a^Live birth rates are five years prior to vaccination observation year (e.g.: 1993 live birth rates for 1998 when child would be five years old)

### MMR scandal

In our cohort, consistent with previous reports [21–24], we observed that the MMR vaccine rates in the UK declined considerably from 93% to 87% between 1993 and 1998 to 1999 and 2004 following the retracted report linking the MMR vaccine to autism [6].

**Fig. 2:**
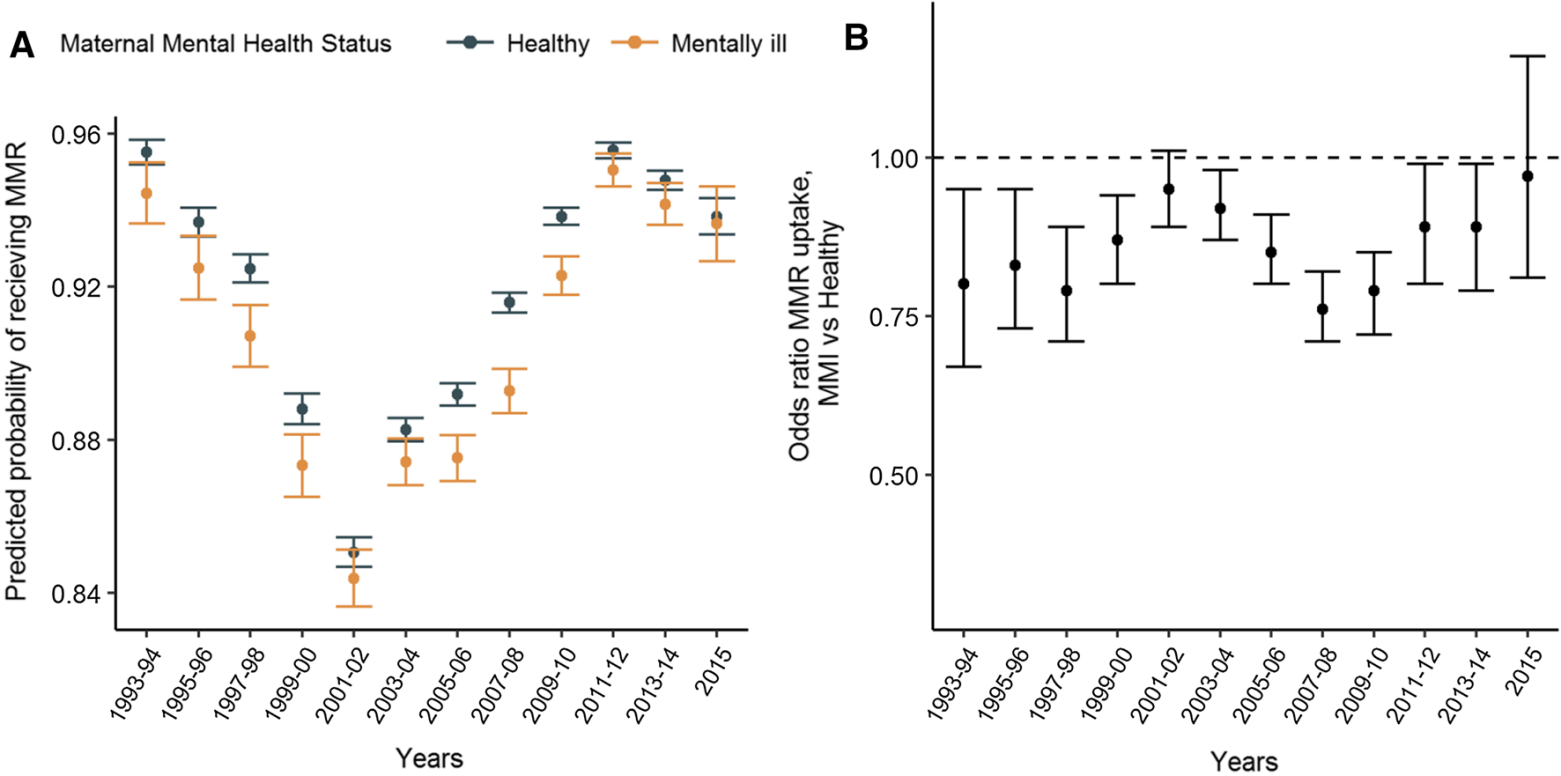
**a** Predicted probability of receiving MMR vaccine by maternal mental illness and year. **b** Association between MMR vaccine uptake and maternal mental illness by year

The rate of MMR vaccination fell similarly in both MMI and healthy groups (Fig. 2a). Some differences were observed: the reduction in MMR uptake was more pronounced following the scandal among children with healthy mothers compared to those with MMI; such that, those with MMI were more similar to those without during the period 1999–2004 than they were prior to 1998 (Fig. 2b). After 2003, the rate improved for both groups, however the improvement appears slower for children with MMI (Fig. 2a).

### Sensitivity analyses

Excluding children born before 2005, in order to test the ‘QOF effect’, led to a very small decrease in the aORs for children exposed to any MMI at two years (supplementary Table 1). The most distinct change was observed for the children exposed to maternal psychotic disorder: as the odds ratio decreased from 0.86 to 0.79. Excluding late registered children did not change the effect estimates (see supplementary Table 2).

## Discussion

### Principal findings

We have conducted the largest contemporary cohort study to-date examining the association between MMI and childhood vaccination uptake in a primary care cohort of over 400,000 mother-baby pair. This study demonstrates that children with MMI are significantly less likely to receive preventative health vaccinations during the first five years of life.

Children exposed to maternal psychotic or depressive disorders had 14% lower likelihood of receiving necessary vaccinations at two years compared to children of well-women. There was some variability in likelihood of vaccination uptake according to type of MMI: children exposed to maternal eating disorders had only 6% lower likelihood of receiving complete vaccination doses when compared to children with healthy mothers and children exposed to maternal substance and alcohol abuse who had a 50% decrease.

We confirm previous reports: over the study period, there was a significant reduction in MMR vaccination uptake in children of both well and mentally ill mothers. The trends were similar; although we showed some evidence that the reduction was slower amongst children with MMI than with healthy mothers, and that the recovery was also slower. We conducted additional analysis to validate these findings by investigating the trends for girls and boys. This revealed that the reduction in MMR uptake was greater for boys, possibly because parents harbour greater fear about risk of autism in boys (see Supplementary Table 3).

### Strengths and limitations

The large sample size of this study allows for precision in the estimates and for us to examine the relationship by type of mental illness and by calendar period. Moreover, using administrative data eliminates the possibility of response or recall bias associated with previous studies using self-reported data.

However, there remain some limitations. The effect of MMI on the vaccination uptake in children may be underestimated for several reasons. First, we used only medical records of the mothers recorded by the GP; and mental illnesses may be under-reported in primary care [35]. This might have led us to misclassify some mentally ill mothers as healthy [14, 36, 37]. This is more likely to influence estimates for children of mothers with less severe mental illness which may be more likely to remain hidden; and also to underestimate effects in non-white mothers and migrant mothers who are less likely to use primary and preventative health services [38, 39]. Also, it was not possible to differentiate between potential unstable mental illness diagnoses and true co-morbidities of mental illnesses in the dataset—if a diagnosis were to have changed over time this would not be distinguished from a comorbid illness. Secondly, in order to capture MMI before the vaccination date, we excluded mothers who did not register to their participating practice at least one year prior to birth. More severe mental illness might be associated with moving-house and moving GP more frequently [40]; therefore, excluding these mothers might have underestimated the effect of MMI because the exclusion might have biased the sample to less severe MMI. Finally, we were not able to capture other potential confounders such as mother’s education level, marital status, social support and parenting practices as these are poorly, or not routinely, recorded in primary care. This is an important gap in our knowledge and would warrant investigation in future studies [41].

### Comparison with previous studies

Comparability with other studies examining MMI and vaccination uptake is limited, either because they were undertaken in different countries with different health systems; and using different exposure definitions; or with different analysis design or different study periods. Existing evidence heavily focuses on common mental disorder. For instance, Turner et al. [11], in a small sample of 159 infants, reported children of mothers with depression were more almost five times more likely to be vaccinated late or not at all (OR 4.92, 95% CI 1.39–17.39). Similarly, Minkovitz et al. [12] reported 21% reduced likelihood of vaccination rate among two year-olds with depressed mothers (OR 0.79, 95% CI 0.69–0.93); this is comparable to our finding of a 15% decrease in the likelihood of vaccination uptake among children aged two. Marginal decreased vaccination uptake among UK infants of mothers with psychotic disorder was reported in 2003 by Howard et al. [13] [relative risk (RR) 0.94, 95% CI 0.88–0.99]; in our study, we followed children through school age (five years). All these studies demonstrate a negative association between maternal mental illness and offspring vaccine uptake.

This is the first time the effects of the MMR scandal has been investigated in a large cohort study. Our findings show that MMR uptake in children of healthy mothers dropped post 1998, reaching the same level as that of mentally ill mothers before 1998. This adds to the growing body of evidence that public health information is not accessed in the same way by people with mental illness [7, 8]; in this case, lack of engagement with misleading public health information may have protected some children in our cohort.

Our results are independent of potential measurable confounders of sex of the child and ethnicity, delivery year, maternal age, practice level deprivation quintile and region. We note that our estimates do not imply a causal link and may be subject to residual confounding. We have not been able to examine further mechanisms explaining the link between MMI and decreased childhood vaccinations. Existing literature suggests that severity of mental illness symptoms could influence a mother’s parenting skills [42] and her decision making with respect to their children’s preventative health-care [12].

### Public health implications

Overall, our results suggest that MMI is associated with a reduced likelihood of vaccination uptake in offspring. Poor maternal mental health has wider cost and resource implications for public health interventions such as the Healthy Child Programme [43] which seeks to support families and children to access preventive public health services through parental advice and accessible information; but it does not explicitly link MMI with a lack of uptake of preventive health care. Neither does the current Healthy Child Programme [43] include tailored approaches to mothers with specific mental illnesses.

Our results explicitly link MMI to illness prevention in children and demonstrate how important this link may be for child health. Children with MMI represent an easily identifiable group of children at risk of not receiving preschool vaccination; while screening of women for mental illness antenatally and postnatally is now part of routine antenatal and postnatal primary care. Public health policies and practice guidelines including the Healthy Child Programme [43] should be modified to target mothers with mental illness. For instance, children exposed to maternal alcohol and substance misuse were at the greatest risk of not receiving necessary vaccinations; therefore, expanding preventive programmes to target these mothers is another clear implication of our findings. Moreover, children exposed to any MMI at two years remained at risk of not receiving vaccinations at five years which suggests that screening and support for MMI is required beyond the postnatal period. Future research should examine whether the disparity in vaccine uptake in pre-school continues throughout childhood, using large population level data.

Our findings also imply that, as is the case for information about smoking cessation [7], public health information about vaccination needs to be personalised to women with mental illness who become mothers. Such a targeted approach could lead to thousands of additional children receiving their necessary vaccinations resulting in significant health and economic benefits. These results are timely following the secretary of state’s recent invocation for a National Health Services (NHS) which is more focussed on preventive health, of which childhood vaccination uptake must play a role [44].

## Conclusions

This study provides essential evidence for policy makers, service planners, commissioners and GPs. It suggests that there is a current and urgent need to improve vaccination uptake among children exposed to MMI. Importantly, this research informs GPs and health visitors that children with MMI are at particular risk of starting school vulnerable to preventable diseases; and that these families are likely to require extra monitoring and support to reverse this health inequality.

## Electronic supplementary material

Below is the link to the electronic supplementary material.

Supplementary material 1 (DOCX 39 kb)

## References

[1] Roush SW, Murphy TV (2007). Group and the V-PDTW Historical comparisons of morbidity and mortality for vaccine-preventable diseases in the United States. JAMA.

[2] World Health Organization. Global Immunization Coverage [Internet]. [cited 2018 Sep 20]. http://www.who.int/news-room/fact-sheets/detail/immunization-coverage.

[3] European Centre for Disease Prevention and Control. Communicable Disease Threats Report [Internet]. [cited 2018 Sep 20]. https://ecdc.europa.eu/sites/portal/files/documents/CDTR_14July2018.pdf.

[4] Public Health England. Measles Outbreak in England [Internet]. 2018 [cited 2018 Aug 27]. https://www.gov.uk/government/news/measles-outbreaks-across-england.

[5] Centers for Disease Control and Prevention. Measles Cases and Outbreaks [Internet]. 2019 [cited 2019 Mar 12]. https://www.cdc.gov/measles/cases-outbreaks.html.

[6] Wakefield AJ, Murch SH, Anthony A, Linnell J, Casson DM, Malik M (1998). RETRACTED: Ileal-lymphoid-nodular hyperplasia, non-specific colitis, and pervasive developmental disorder in children. Lancet.

[7] Webb RT, Wicks S, Dalman C, Pickles AR, Appleby L, Mortensen PB (2010). Influence of environmental factors in higher risk of sudden infant death syndrome linked with parental mental illness. Arch Gen Psychiatry.

[8] Banham L, Gilbody S (2010). Smoking cessation in severe mental illness: what works?. Addiction.

[9] Gross GJ, Howard M (2001). Mothers’ decision-making processes regarding health care for their children. Public Health Nurs.

[10] Abel KM, Hope H, Swift E, Parisi R, Ashcroft DM, Kosidou K (2019). Prevalence of maternal mental illness among children and adolescents in the UK between 2005 and 2017: a national retrospective cohort analysis. Lancet Public Health.

[11] Turner C, Boyle F, O’Rourke P (2003). Mothers’ health post-partum and their patterns of seeking vaccination for their infants. Int J Nurs Pract.

[12] Minkovitz CS, Strobino D, Scharfstein D, Hou W, Miller T, Mistry KB (2005). Maternal depressive symptoms and children’s receipt of health care in the first 3 years of life. Pediatrics.

[13] Howard LM, Goss C, Leese M, Thornicroft G (2003). Medical outcome of pregnancy in women with psychotic disorders and their infants in the first year after birth. Br J Psychiatry.

[14] Ban L, Gibson JE, West J, Fiaschi L, Oates MR, Tata LJ (2012). Impact of socioeconomic deprivation on maternal perinatal mental illnesses presenting to UK general practice. Br J Gen Pract.

[15] Webb R, Abel K, Pickles A, Appleby L (2005). Mortality in offspring of parents with psychotic disorders: a critical review and meta-analysis. Am J Psychiatry.

[16] Webb RT, Abel KM, Pickles AR, Appleby L, King-Hele SA, Mortensen PB (2006). Mortality risk among offspring of psychiatric inpatients: a population-based follow-up to early adulthood. Am J Psychiatry.

[17] Herrett E, Gallagher AM, Bhaskaran K, Forbes H, Mathur R, van Staa T (2015). Data resource profile: Clinical Practice Research Datalink (CPRD). Int J Epidemiol.

[18] Chisholm J (1990). The read clinical classification. BMJ.

[19] Margulis AV, Abou-Ali A, Strazzeri MM, Ding Y, Kuyateh F, Frimpong EY (2013). Use of selective serotonin reuptake inhibitors in pregnancy and cardiac malformations: a propensity-score matched cohort in CPRD. Pharmacoepidemiol Drug Saf.

[20] Clinical Practice Research Datalink (CPRD) (2017). Mother–baby link documentation.

[21] NHS Digital. Childhood Vaccination Coverage Statistics [Internet]. 2017 [cited 2018 Oct 21]. https://digital.nhs.uk/data-and-information/publications/statistical/nhs-immunisation-statistics/england-2017-18.

[22] ISD Scotland. Childhood Immunisation Statistics Scotland [Internet]. [cited 2018 Oct 21]. http://www.isdscotland.org/Health-Topics/Child-Health/Immunisation/.

[23] Public Health Wales. Surveillance Data for Vaccine Preventable Diseases in Wales [Internet]. 2018 [cited 2018 Oct 21]. http://www.wales.nhs.uk/sites3/page.cfm?orgid=457&pid=27777.

[24] Public Health Agency. Northern Ireland Vaccination Coverage [Internet]. 2018 [cited 2018 Oct 21]. http://www.publichealth.hscni.net/directorate-public-health/health-protection/vaccination-coverage.

[25] Rait G, Walters K, Griffin M, Buszewicz M, Petersen I, Nazareth I (2009). Recent trends in the incidence of recorded depression in primary care. Br J Psychiatry.

[26] Public Health England. An estimated one in seven 5 year olds not immunised against MM [Internet]. 2019 [cited 2020 Jan 14]. https://www.gov.uk/government/news/an-estimated-one-in-seven-5-year-olds-not-immunised-against-mmr.

[27] NHS England. Scheduling and timing of pre-school booster vaccinations [Internet]. 2016 [cited 2020 Jan 14]. https://www.england.nhs.uk/south/wp-content/uploads/sites/6/2016/07/schedul-timing-pre-schl-boost.pdf.

[28] Saunders CL, Abel GA, El Turabi A, Ahmed F, Lyratzopoulos G (2013). Accuracy of routinely recorded ethnic group information compared with self-reported ethnicity: evidence from the English Cancer Patient Experience survey. BMJ Open.

[29] National Records of Scotland. Births Time Series Data [Internet]. 2017 [cited 2018 May 25]. https://www.nrscotland.gov.uk/statistics-and-data/statistics/statistics-by-theme/vital-events/births/births-time-series-data.

[30] Office for National Statistics. Births in England and Wales: 2017 [Internet]. [cited 2018 May 25]. https://www.ons.gov.uk/peoplepopulationandcommunity/birthsdeathsandmarriages/livebirths/bulletins/birthsummarytablesenglandandwales/2017.

[31] Northern Ireland Statistics and Research Agency. Birth Statistics [Internet]. 2018 [cited 2018 May 25]. https://www.nisra.gov.uk/publications/birth-statistics.

[32] Woodward M (2013). Epidemiology: study design and data analysis.

[33] Williams RL (2000). A note on robust variance estimation for cluster-correlated data. Biometrics.

[34] Mathur R, Bhaskaran K, Chaturvedi N, Leon DA, Van Staa T, Grundy E (2014). Completeness and usability of ethnicity data in UK-based primary care and hospital databases. J Public Health.

[35] Sharma VK, Copeland JRM (2009). Detecting mental disorders in primary care. Ment Health Fam Med.

[36] Baker R, Kendrick D, Tata LJ, Orton E (2017). Association between maternal depression and anxiety episodes and rates of childhood injuries: a cohort study from England. Inj Prev.

[37] Ban L, Gibson JE, West J, Tata LJ (2010). Association between perinatal depression in mothers and the risk of childhood infections in offspring: a population-based cohort study. BMC Public Health.

[38] Forster AS, Rockliffe L, Chorley AJ, Marlow LAV, Bedford H, Smith SG (2017). Ethnicity-specific factors influencing childhood immunisation decisions among Black and Asian Minority Ethnic groups in the UK: a systematic review of qualitative research. J Epidemiol Community Health.

[39] Smith MS, Lawrence V, Sadler E, Easter A (2019). Barriers to accessing mental health services for women with perinatal mental illness: systematic review and meta-synthesis of qualitative studies in the UK. BMJ Open.

[40] Phinney R (2013). Exploring residential mobility among low-income families. Soc Serv Rev.

[41] Abel KM, Newbigging K (2018). Addressing unmet needs in women’s health.

[42] Rampou AM, Havenga Y, Madumo M (2015). Parenting experiences of mothers living with a chronic mental illness. Health SA Gesondheid.

[43] Department of Health. Healthy Child Programme-Pregnancy and the first 5 years of life [Internet]. 2009 [cited 2018 Mar 31]. https://assets.publishing.service.gov.uk/government/uploads/system/uploads/attachment_data/file/167998/Health_Child_Programme.pdf.

[44] Department of Health and Social Care. Prevention is better than cure [Internet]. London; 2018 [cited 2018 Nov 7]. https://assets.publishing.service.gov.uk/government/uploads/system/uploads/attachment_data/file/753688/Prevention_is_better_than_cure_5-11.pdf.

