# Supplementary material for: The influence of maternal mental illness on vaccination uptake in children: a UK population-based cohort study
Source: Eur J Epidemiol. 2020 Apr 24;35(9):879–89. doi: 10.1007/s10654-020-00632-5 (PMC7524844; doi:10.1007/s10654-020-00632-5)
Supplement: Supplementary file 1 — Supplementary material 1 (DOCX 39 kb) [file 10654_2020_632_MOESM1_ESM.docx]

# SUPPLEMENTARY TABLES

## Supplementary ****Table 1-**** Sensitivity Analysis examining data prior and after introduction of QOF

| **Up to Date Vaccinations at** | **Primary Analysis Adjusted Model- 1 ^a^** | **Excluding children who were born before 2005** |
| --- | --- | --- |
|  | **(n= 479, 949)** | **(n=283, 921)** |
| **2 Year** |  |  |
| Unexposed to maternal mental illness | **REF** | **REF** |
| Exposed to any maternal mental illness | 0·86 (0·84-0·88) | 0·84 (0·81-0·87) |
|  |  |  |
| Psychotic disorder | 0·86 (0·71-1·03) | 0·79 (0·61-1·01) |
| Depressive disorder | 0·86 (0·84-0·88) | 0·82 (0·80-0·85) |
| Anxiety disorder | 0·86 (0·83-0·89) | 0·87 (0·82-0·92) |
| Eating disorder | 0·94 (0·77-1·16) | 1·11 (0·78-1·58) |
| Personality disorder | 0·75 (0·58-0·98) | 0·82 (0·56-1·20) |
| Substance and alcohol abuse | 0·50 (0·44-0·58) | 0·51 (0·42-0·62) |
|  |  |  |
| **5 Year** |  |  |
| Unexposed to maternal mental illness | **REF** | **REF** |
| Exposed to any maternal mental illness | 0·86 (0·84-0·88) | 0·84 (0·81-0·87) |
|  |  |  |
| Psychotic disorder | 0·71 (0·62-0·82) | 0·67 (0·55-0·83) |
| Depressive disorder | 0·86 (0·84-0·88) | 0·83 (0·80-0·86) |
| Anxiety disorder | 0·84 (0·82-0·87) | 0·82 (0·78-0·86) |
| Eating disorder | 0·83 (0·71-0·98) | 0·84 (0·64-1·11) |
| Personality disorder | 0·64 (0·52-0·78) | 0·55 (0·41-0·73) |
| Substance and alcohol abuse | 0·50 (0·45-0·56) | 0·49 (0·41-0·58) |
|  |  |  |
| **^a^**^:^ Adjusted for sex of the child, child ethnicity delivery year, maternal age, practice level deprivation quintile and region. | | |

## ****Supplementary Table 2-** Sensitivity analysis examining the effect of late registered children**

| **Up to Date Vaccinations at** | **Primary Analysis Adjusted Model- 1^a^** | **Excluding late registered children** |
| --- | --- | --- |
|  | **(n= 479, 949)** | **(n=474, 010)** |
| **2 Year** |  |  |
| Unexposed to maternal mental illness | **REF** | **REF** |
| Exposed to any maternal mental illness | 0·86 (0·84-0·88) | 0·86 (0·84-0·88) |
|  |  |  |
| Psychotic disorder | 0·86 (0·71-1·03) | 0·83 (0·69-1·00) |
| Depressive disorder | 0·86 (0·84-0·88) | 0·86 (0·84-0·88) |
| Anxiety disorder | 0·86 (0·83-0·89) | 0·86 (0·83-0·89) |
| Eating disorder | 0·94 (0·77-1·16) | 0·96 (0·78-1·17) |
| Personality disorder | 0·75 (0·58-0·98) | 0·76 (0·58-0·99) |
| Substance and alcohol abuse | 0·50 (0·44-0·58) | 0·49 (0·43-0·57) |
|  |  |  |
| **5 Year** |  |  |
| Unexposed to maternal mental illness | **REF** | **REF** |
| Exposed to any maternal mental illness | 0·86 (0·84-0·88) | 0·86 (0·84-0·88) |
|  |  |  |
| Psychotic disorder | 0·71 (0·62-0·82) | 0·70 (0·61-0·81) |
| Depressive disorder | 0·86 (0·84-0·88) | 0·85 (0·33-0·87) |
| Anxiety disorder | 0·84 (0·82-0·87) | 0·84 (0·81-0·87) |
| Eating disorder | 0·83 (0·71-0·98) | 0·84 (0·72-0·99) |
| Personality disorder | 0·64 (0·52-0·78) | 0·62 (0·50-0·77) |
| Substance and alcohol abuse | 0·50 (0·45-0·56) | 0·50 (0·45-0·56) |
|  |  |  |
| **^a:^** Adjusted for sex of the child, child ethnicity delivery year, maternal age, practice level deprivation quintile and region | | |

## ****Supplementary Table 3-** Odds Ratio of girls receiving MMR vaccine uptake compared to boys in years**

|  | **Adjusted Model 1^a^** | |
| --- | --- | --- |
| **Years** | **aOR (95%Cl)** | **P value** |
| 1993-94 | 0·96 (0·84-1·09) | 0·535 |
| 1995-96 | 1·02 (0·92-1·14) | 0·695 |
| 1997-98 | 1·05 (0·96-1·15) | 0·303 |
| 1999-00 | 1·17 (1·09-1·23) | <0·001 |
| 2001-02 | 1·16 (1·10-1·22) | <0·001 |
| 2003-04 | 1·08 (1·02-1·14) | 0·005 |
| 2005-06 | 1·11 (1·05-1·17) | <0·001 |
| 2007-08 | 1·09 (1·03-1·15) | <0·001 |
| 2009-10 | 1·02 (0·96-1·09) | 0·486 |
| 2011-12 | 1·06 (0·98-1·15) | 0·168 |
| 2013-14 | 1·13 (1·03-1·23) | 0·012 |
| 2015 | 1·20 (1·03-1·39) | 0·018 |
| **^a:^** Adjusted for child ethnicity delivery year, maternal age, practice level deprivation quintile and region | | |

## Supplementary Table 4- Odds Ratio of receiving vaccinations at age two among children with and without mentally ill mothers: adjusted variables

|  | **Adjusted Model 1** | | **Adjusted Model 2** | |
| --- | --- | --- | --- | --- |
| **Up to Date Vaccinations at** | **OR (95% Cl)** | ***P* value** | **OR (95% Cl)** | ***P* value** |
| **2 Year** |  |  |  |  |
| Unexposed to maternal mental illness | **REF** |  | **REF** |  |
| Exposed to any maternal mental illness | 0·86 (0·84-0·88) | <0·001 | 0·86 (0·84-0·88) | <0.001 |
| Child Sex (Female) | 1·07 (1·06-1·09) | <0·001 | 1·07 (1·06-1·09) | <0.001 |
| Delivery Year | 1·09 (1·08-1·09) | <0·001 | 1·08 (1·08-1·09) | <0.001 |
| Delivery Year^2 | 1·01 (1·00-1·01) | <0·001 | 1·01 (1·00-1·01) | <0.001 |
| Maternal Age | 1·00 (1·00-1·00) | <0·001 | 1·00 (1·00-1·00) | <0.001 |
| Maternal Age^2 | 1·00 (1·00-1·00) | <0·001 | 1·00 (1·00-1·00) | <0.001 |
| Prenatal GP Consultation | - | - | 1·02 (1·01-1·02) | <0·001 |
|  |  |  |  |  |
| **Ethnicity** |  |  |  |  |
| White | **REF** |  | **REF** |  |
| Asian/ British Asian | 1·26 (1·18-1·34) | <0·001 | 1·25 (1·17-1·34) | <0·001 |
| Black/Black British | 0·84 (0·78-0·91) | <0·001 | 0·84 (0·78-0·91) | <0·001 |
| Mixed | 0·84 (0·78-0·90) | <0·001 | 0·84 (0·78-0·90) | <0·001 |
| Other | 1·02 (0·92-1·13) | 0·727 | 1·02 (0·92-1·12) | 0·769 |
| Unknown | 0·77 (0·75-0·79) | <0·001 | 0·77 (0·75-0·79) | <0·001 |
|  |  |  |  |  |
| **UK IMD Quintile** *(Based on GP Location)* |  |  |  |  |
| 1 (least deprived) | **REF** |  |  |  |
| 2 | 0·93 (0·89-0·96) | <0·001 | 0·93 (0·90-0·96) | <0·001 |
| 3 | 0·92 (0·89-0·95) | <0·001 | 0·92 (0·89-0·96) | <0·001 |
| 4 | 0·87 (0·85-0·90) | <0·001 | 0·87 (0·85-0·91) | <0·001 |
| 5 (most deprived) | 0·80 (0·78-0·83) | <0·001 | 0·81 (0·87-0·83) | <0·001 |
|  |  |  |  |  |
| **Region** |  |  |  |  |
| South Central | **REF** |  | **REF** |  |
| North East | 1·01 (0.93-1.09) | 0·830 | 1·02 (0·94-1·11) | 0·578 |
| North West | 0·93 (0.89-0.97) | 0·002 | 0·94 (0·90-0·98) | 0·008 |
| Yorkshire & The Humber | 1·08 (1.01-1.14) | 0·017 | 1·09 (1·02-1·16) | 0·007 |
| East Midlands | 1·33 (1.25-1.41) | <0·001 | 1·34 (1·26-1·43) | <0·001 |
| West Midlands | 0·99 (0.94-1.04) | 0·633 | 1·00 (0·95-1·05) | 0·927 |
| East of England | 0·90 (0.86-0.94) | <0·001 | 0·91 (0·87-0·95) | <0·001 |
| South West | 0·88 (0.84-0.93) | <0·001 | 0·89 (0·85-0·93) | <0·001 |
| London | 0·57 (0.54-0.59) | <0·001 | 0·58 (0·55-0·60) | <0·001 |
| South East Coast | 0·83 (0.79-0.87) | <0·001 | 0·84 (0·80-0·88) | <0·001 |
| Northern Ireland | 0·90 (0.85-0.95) | <0·001 | 0·90 (0·85-0·96) | <0·001 |
| Scotland | 0·95 (0.90-0.99) | 0·029 | 0·95 (0·91-1·00) | 0·052 |
| Wales | 0·81 (0.78-0.85) | <0·001 | 0·82 (0·78-0·86) | <0·001 |

## Supplementary Table 5- Odds Ratio of receiving vaccinations at age five among children with and without mentally ill mothers: adjusted variables

|  | **Adjusted Model 1** | | **Adjusted Model 2** | |
| --- | --- | --- | --- | --- |
| **Up to Date Vaccinations at** | **OR (95% Cl)** | ***P* value** | **OR (95% Cl)** | **P value** |
| **5 Year** |  |  |  |  |
| Unexposed to maternal mental illness | **REF** |  | **REF** |  |
| Exposed to any maternal mental illness | 0·80 (0·84-0·88) | <0·001 | 0·85 (0·84-0·87) | <0·001 |
| Child Sex (Female) | 1·07 (1·05-1·09) | <0·001 | 1·07 (1·05-1·09) | <0·001 |
| Delivery Year | 1·11 (1·11-1·12) | <0·001 | 1·11 (1·11-1·12) | <0·001 |
| Delivery Year^2 | 1·01 (1·01-1·01) | <0·001 | 1·01 (1·01-1·01) | <0·001 |
| Maternal Age | 1·00 (1·00-1·00) | <0·001 | 1·00 (1·00-1·00) | <0·001 |
| Maternal Age^2 | 1·00 (1·00-1·00) | <0·001 | 1·00 (1·00-1·00) | <0·001 |
| Prenatal GP Consultation | - | - | 1·02 (1·02-1·03) | <0·001 |
|  |  |  |  |  |
| **Ethnicity** |  |  |  |  |
| White | **REF** |  | **REF** |  |
| Asian/ British Asian | 1·14 (1·07-1·22) | <0·001 | 1·13 (1·06-1·21) | <0·001 |
| Black/Black British | 0·84 (0·77-0·92) | <0·001 | 0·83 (0·76-0·91) | <0·001 |
| Mixed | 0·80 (0·74-0·87) | <0·001 | 0·80 (0·73-0·86) | <0·001 |
| Other | 0·99 (0·88-1·10) | 0.840 | 0·99 (0·88-1·10) | 0.799 |
| Unknown | 0·80 (0·78-0·82) | <0·001 | 0·81 (0·79-0·83) | <0·001 |
|  |  |  |  |  |
| **UK IMD Quintile** *(Based on GP Location)* |  |  |  |  |
| 1 (least deprived) | **REF** | **-** | **REF** |  |
| 2 | 0·96 (0·93-1·00) | 0·034 | 0·96 (0·93-1·00) | 0.053 |
| 3 | 0·94 (0·91-0·97) | <0·001 | 0·94 (0·91-0·98) | 0.001 |
| 4 | 0·88 (0·86-0·91) | <0·001 | 0·89 (0·86-0·92) | <0·001 |
| 5 (most deprived) | 0·76 (0·74-0·79) | <0·001 | 0·76 (0·74-0·79) | <0·001 |
|  |  |  |  |  |
| **Region** |  |  |  |  |
| South Central | **REF** |  | **REF** |  |
| North East | 1·15 (1·06-1·25) | 0·001 | 1·17 (1·08-1·27) | <0·001 |
| North West | 0·98 (0·94-1·03) | 0·425 | 0·99 (0·95-1·04) | 0·807 |
| Yorkshire & The Humber | 1·00 (0·94-1·06) | 0·970 | 1·01 (0·95-1·08) | 0·711 |
| East Midlands | 1·38 (1·30-1·47) | <0·001 | 1·40 (1·32-1·49) | <0·001 |
| West Midlands | 1·08 (1·03-1·13) | 0·002 | 1·09 (1·04-1·15) | <0·001 |
| East of England | 0·92 (0·88-0·96) | <0·001 | 0·93 (0·89-0·98) | 0·003 |
| South West | 1·01 (0·97-1·07) | 0·572 | 1·02 (0·97-1·07) | 0·476 |
| London | 0·56 (0·53-0·58) | <0·001 | 0·57 (0·54-0·59) | <0·001 |
| South East Coast | 0·91 (0·86-0·95) | <0·001 | 0·92 (0·87-0·96) | <0·001 |
| Northern Ireland | 1·09 (1·02-1·16) | 0·007 | 1·09 (1·03-1·16) | 0·006 |
| Scotland | 1·11 (1·06-1·17) | <0·001 | 1·12 (1·07-1·18) | <0·001 |
| Wales | 0·88 (0·84-0·92) | <0·001 | 0·88 (0·84-0·93) | <0·001 |
